# Hazelnut skins as a new sustainable ingredient for beef cattle diets

**DOI:** 10.1038/s41598-025-22896-1

**Published:** 2025-11-06

**Authors:** Elena Diaz Vicuna, Edoardo Fiorilla, Karthika Srikanthithasan, Valeria Zambotto, Chiara Bianchi, Fulvio Riondato, Manuela Renna, Rosangela Odore, Paola Badino, Giulia Gardini, Valentina Arneodo, Andrea Cravero, Laura Ozella, Andrea Giorgino, Silvia Tabasso, Giorgio Capaldi, Alberto Brugiapaglia, Claudio Forte

**Affiliations:** 1 Department of Veterinary Sciences, University of Turin, Grugliasco, Italy; 2 Department of Drug Science and Technology, University of Turin, Turin, Italy; 3 Department of Agriculture, Forest and Food Sciences, University of Turin, Grugliasco, Italy

**Keywords:** Livestock, Food by-product, Re-evaluation, *In vivo*, Alternative feed material, Hazelnut skins, Zoology, Environmental sciences

## Abstract

Hazelnut skins (HS), by-products of the hazelnut industry classified as waste, are rich in polyphenols, poli-unsaturated fatty acids and fibres. This study aimed at assessing the *in vivo* effects of HS inclusion in beef cattle diets. Eighty beef bulls were divided in two groups (control and test; 4 replicates per group), following a two-phase dietary plan (fattening – 5 months; finishing – 2 months). Both groups received forage (hay/straw) and feed, with the test feed presenting an 8% HS inclusion. Feeds’ chemical composition and total phenolic contents were assessed. Bulls’ *in vivo* growth performances were recorded, and haematological analysis were conducted at trial start and on slaughter day. Test feeds of both phases presented higher values (p < 0.001) of polyphenols than the control ones. No differences were observed in haematological or growth parameters during the fattening phase. In the finishing, the control group exhibited a lower (p < 0.05) feed conversion ratio than the test. However, the potential health benefits linked to the HS’ phenolic compounds, along with the re-valorization of an agro-industrial by-product and the resulting reduction in food waste, support the hypothesis that including HS in beef cattle diets may constitute a viable and sustainable strategy for the beef industry.

**Supplementary Information:**

The online version contains supplementary material available at 10.1038/s41598-025-22896-1.

## Introduction

The demand for beef meat in Europe has been characterized by a slow, yet steady decline over recent years. The trend is probably the result of growing consumer concern surrounding the impact of cattle farming in terms of natural resources and economic factors as well as human and animal health and welfare^1,2^. To align the realities of the beef industry with consumers’ evolving expectations, a key challenge for the sector will necessarily involve the implementation of broader sustainability practices in addition to those associated with productivity.

Feed is a major driver of the industry’s economic and environmental costs. By consequence, research in alternative ingredients, such as insects, marine algae and food industry by-products, is also growing^3,4^. Food by-products, generated during the various phases of agricultural and food processing operations, are one of the most promising solutions being investigated, as they offer a rich source of micro-nutrients and bioactive compounds^5^. Different sub-groups of by-products exist, reflecting their different origins, nutritional profiles and possible uses. Agro-industrial by-products include dairy wastes, fruit peels and pulps, vegetable trimmings and husks from certain seeds and fruits^6–9^.

The hazelnut industry holds a significant position in Italy, not only in economic terms, being the second biggest producer world-wide, but also for its historical presence in certain regions, such as Piedmont, Latium and Campania^10^. The processing of hazelnuts results in various by-products, including the leaves, green leaf coverings, shells and skins^11^. Hazelnut skins (HS) are identified as the brown perisperm tightly attached to the fruit, which naturally separates from the latter during the roasting process^11^. In Italy, approximately 3000 tons of roasted HS are produced annually and discarded as waste, representing both an economic and ecological challenge for the sector^12^. HS are the only element of the hazelnut supply chain generated during the post-harvesting phase classified as “waste”. Thus, re-evaluating their use opens the possibilities to make the hazelnut industry fully sustainable, creating a “cradle-to-cradle” industrial system.

The chemical profile of HS is characterized by a low moisture content (~ 7%) and a total dietary fibre content of approximately 69%, much higher than that of the hazelnut itself (not exceeding 14% on a dry matter basis)^13,14^. Furthermore, HS contain high amounts of fatty acids, such as oleic, linoleic and palmitic acids, at percentages of 76%, 15% and 6%, respectively, on a dry matter basis^15^. The HS are also rich in bioactive compounds, especially phenols, present in concentrations 168 to 378 higher than in raw hazelnuts^16,17^. Numerous studies have demonstrated the beneficial effects of HS polyphenols in promoting well-being and preventing chronic diseases thanks to their antioxidant, anti-inflammatory and hypolipidemic properties derived from the chemical profile of HS^18^. Moreover, these compounds have exhibited in vitro antibacterial properties against a wide range of pathogenic and saprophytic bacteria^19,20^. On this basis, the consumption of HS has the potential to reduce antibiotic use in livestock farming, in line with European strategies against antimicrobial resistance^21^. Although evidence on their *in vivo* efficacy when supplied as a feed ingredient is still lacking, efforts to fill this gap are currently being explored, with studies reporting promising preliminary *in vivo* and *postmortem* results, in both monogastrics (pigs) and ruminants (dairy cows, lactating ewes, beef lambs)^11,22–24^. Considering the findings reported in these studies, we can hypothesize HS to be a sustainable and safe feed ingredient to use for beef cattle that could help meet the growing demand for a more sustainable livestock sector. Despite the high-profile beef industry in Italy and its leading position in global hazelnut production, no studies have yet investigated the aforementioned potentialities.

To address this gap, the LIVE-HAZE project was developed, aimed at evaluating the effects of HS dietary inclusion in different livestock species. In particular, the present study was designed to evaluate the effects of HS in beef cattle in terms of *in vivo* and *postmortem* performance, health indicators, meat quality and sensory characteristics, alongside the economic and environmental implications for both the beef and hazelnut supply chains. Thus, the aim of the present paper was to gain insight into the effects of HS inclusion on the oxidative stability of the feed, and on the animals’ *in vivo* performance and health status, assessed through haematological analyses and MALDI-TOF–MS serum proteomic profiling. Our findings are expected to support the repurposing of a current by-product of the hazelnut industry, ultimately contributing towards improving the overall sustainability of the livestock industry.

## Methods

### The trial (animals, experimental design dietary treatments)

The experimental trial took place on a beef commercial farm located in Rivoli (TO) (North-West Italy) and was conducted from June 2023 to October 2024. The experimental procedures with animals were conducted in accordance with European Union legislation for the protection of animals used for experimental and other scientific purposes^25^, being approved by the Bioethical Committee of the University of Turin, Italy, (protocol no. 0513042, 03/10/2022), and conducted in accordance with ARRIVE guidelines. Through a randomized block design, a total of 80 Piemontese beef bulls aged 8 ± 2 months and with an average initial body weight (BW) of 282 ± 65 kg were assigned to two dietary groups (control – CTR, test – T, n = 40 per group).Bulls were grouped according to their initial BW, and each group comprised 4 replicates of 10 animals each. Each replicate was housed in a box measuring 6 × 6 m equipped with 2 drinkers and a feeding alley.

The animals were introduced into the trial gradually over a six-month period (Table 1) and in pairs of replicates belonging to the two dietary treatments. Before the start of the trial, each animal was vaccinated against the main bovine infectious agents (IBR, BVD-MD, PI3, VRSB) and de-wormed. At the end of the trial period, all animals in study were slaughtered in the commercial slaughterhouse belonging to the same farm where the trial was conducted. The slaughterhouse was CE approved, and the slaughtering was conducted in accordance with EC Regulation 1099/2009^26^ on the protection of animals at the time of killing.

**Table 1:** Trial introduction dates of the 4 pairs of control-test replicates involved in the study.

Replicates	Trial start date (dd/mm/yyyy)
CTR1 – T1	01/06/2023
CTR2 – T2	20/07/2023
CTR3 – T3	13/10/2023
CTR4 – T4	21/12/2023

CTR – control group; T – test group; the identity number assigned to each replicate (n.1–2–3–4) correspond to their order of introduction into the trial.

Both the CTR and T groups were fed iso-energetic and iso-nitrogenous diets, with a feeding plan characterized by a 5-month fattening phase followed by a 2-month finishing period.

The diets consisted of a forage fraction (a morning hay ration + an evening straw ration) plus a concentrate component (fattening or finishing feed, depending on the trial phase). The overall dietary plan was structured according to the quantities reported in Table 2, while the ingredients of the fattening and finishing feeds (CTR and T) are listed in Supplementary Material (Table S1). The nutritional plans were formulated in accordance with the general NRC guidelines^27^ and tailored according to the specific requirements of the Piemontese breed, under the supervision of a nutritionist in coordination with the Consorzio di Tutela della Razza Piemontese (COALVI)^28^. Fattening and finishing concentrates for both groups were prepared on farm every two weeks.

**Table 2:** Individual dietary plans (kg/day/animal) for the experimental groups during the fattening and finishing phases.

Ration composition	Fattening diet (kg/day/animal)	Finishing diet (kg/day/animal)
Barley straw	1	2
Hay	2	4
Fattening feed	7.5	
Finishing feed		10

The HS used in both the fattening and finishing feeds for the T group were not subjected to any physical or chemical treatment beyond the industrial roasting process necessary for their detachment from the fruit. Due to the intrinsic fragility of the material, they presented a powder-like texture at the time of inclusion in the feed concentrate.

### Analysis

#### Chemical analysis

Throughout the entire trial, monthly samples of HS and weekly samples of the fattening and finishing feeds (~ 20–30 g/sample) were collected and stored in non-sterile blue-cap jars at 0–4 °C. The representative samples were mixed homogeneously, then finely ground with a mill (mesh or particle size = 1 mm). Chemical composition evaluations were performed in accordance with the guidelines set by the Association of Official Analytical Chemists AOAC^29^, specifically: dry matter (DM, #934.01), ash (#942.05) content, crude protein (CP, Kjeldahl method #984.13) content. The estimation of ether extract (EE) content was carried out through Soxhlet extraction with diethyl ether, following the AOAC International method #2003.05^29^. Neutral detergent fibre (NDF) content was determined according to AOAC method #2002.04, while acid detergent fibre (ADF) content and acid detergent lignin content followed AOAC method #973.18. All samples were analysed in duplicate^30^. The chemical profile of the HS and of the experimental fattening and finishing feeds are summarized in tables S2 and S3 of Supplementary Material, respectively. The chemical composition analysis of HS was characterized by a high EE content (25.03%), along with notable levels of NDF (22.54%) and ADF (17.07%).

#### Oxidative status analysis

Monthly samples of the fattening and finishing feeds were collected throughout the whole trial and stored in non-sterile blue-cap jars at −20 °C. Per each sample, total phenolic content (TPC) and lipid oxidation, measured as thiobarbituric acid reactive substances (TBARS), were determined.

##### Total phenolic content (TPC) analysis

Polyphenol extraction and TPC determination were performed following the standard protocol reported in Capaldi et al.^20^.

- ● Polyphenol extraction

The feed samples were subjected to conventional extraction through hydroalcoholic solution (70:30 EtOH: H2O). The protocol provided for a solid:liquid ratio of 1:30 (1 g in 30 ml), for 60 min at 100 °C. The extracts were filtered, and the ethanol was removed by rotavapor. The remaining aqueous fraction was lyophilized with lyophiliser LyoQuest-85 (Telstar, Madrid, Spain) and conserved at −20 °C.

- ● Analysis of UV-Vis polyphenols

The determination of the TPC was performed following a modified version of the Folin-Ciocalteu method described by Hillis and Swain^31^. For each extract, an aliquot of 0.25 ml was combined with 0.5 ml of a 10% w/v solution of Na2CO3, followed by the addition of 0.25 ml of Folin-Ciocalteu reagent. Distilled water was added in order to produce a final volume of 5 ml, and the final solution left at room temperature and in the dark for 25 min. The solution’s absorbance at 725 nm was measured using a UV–vis Cary 60 spectrophotometer (Agilent Technologies, Santa Clara, CA, USA). Gallic acid was used as reference compound to prepare the calibration curve. The TPC was then expressed in mg gallic acid equivalents (mgGAE) and compared to grams of feed used for extraction (mgGAE/g feed). All tests were performed in triplicate.

##### Lipid oxidation measurement

Lipid oxidation was assessed using the thiobarbituric acid reactive substances (TBARS) assay, following a modified procedure of the method described by Witte, Krause and Bailey^32^.

Briefly, 2 g of frozen feed sample were homogenized for 30 s at high speed in 20 mL of a 10% trichloroacetic acid (TCA) solution using a Polytron tissue homogenizer (Type PT 10–35; Kinematica GmbH, Luzern, Switzerland). The homogenate was subsequently filtered and 1 mL of the resulting filtrate mixed with 1 mL of a 0.02 M aqueous solution of 2-thiobarbituric acid (TBA). The mixture was incubated in a water bath at 100 °C for 20 min, together with a blank containing 1 mL of TCA and 1 mL of TBA solution, and then immediately cooled on ice. Absorbance was measured at 532 nm using a spectrophotometer (UV-1900i, Shimadzu). Analyses were carried out in triplicate, and the results were expressed as g of malondialdehyde (MDA)/kg feed, with a 6-point standard curve as reference, covering the 0.5–10 µM 1,1,3,3-tetramethoxypropane (MDA indicator) concentration range.

#### *In vivo* performance

Individual BW (kg) was recorded at the start of the trial (d_0_), at the time of diet shift (from fattening to finishing) (d_150_), and on the day of slaughter (d_210_). Bi-weekly measurements of forage (hay, straw) and feed (fattening/finishing) intake were performed for each replicate over two consecutive days by weighing the amounts offered and the remaining refused feed before the administration of the next rations.

The individual average daily gain (ADG, kg/d), average individual daily intake of hay (AIDI_H_, kg), straw (AIDI_S_, kg), and feed (AIDI_F_, kg), and feed conversion ratio (FCR, kg/kg; only concentrate feed was considered for calculation) were calculated on the replicate basis for each feeding phase (fattening – t1; finishing – t2) and for the whole experimental period (overall).

#### Climatic data

Climatic data (temperature and humidity) encompassing the years 2023 and 2024 were sourced from the website of the “Agenzia Regionale per la Protezione Ambientale” for Piedmont^33^. The weather station of choice was Torino Alenia (Lat. 45.081441, Long. 7.611727), due to its geographical proximity to the commercial farm where the trial was held and for its being equipped with both a hygrometer and a thermometer. Recorded data encompassed daily minimum, average and maximum temperatures, along with relative humidity.

From the average values, the average climatic temperature – humidity index (THI) value was calculated^34^. This climatic index is used to evaluate the degree of thermal discomfort in both animals and humans, by combining different microclimatic parameters into a single measure^35^.

The THI was calculated using the equation originally developed by^34^:

$$THI= 0.8\cdot T+ RH \cdot (T-14.4) + 46.4$$

where T is the ambient temperature (°C) and RH is the percent relative humidity.

#### Haematological analysis

On d_0_, blood samples were drawn before the administration of the morning feed from the coccygeal vein or jugular vein of all individuals (n = 80) and transferred into individual EDTA tubes (volume 2.5 mL). Right after collection, samples were gently inverted several times and immediately transported to the laboratory for complete blood count (CBC) analysis. Following the same protocol, blood samples were also collected on slaughter day (d_210_) from a representative sample of each dietary group (n = 24/group), selected on the basis of the group’s average age and BW values at d_0_ and d_150_. The CBC analysis, which included a differential leukocyte count, was carried out using an automated haematology analyser (ADVIA® 2120, Siemens, Munich, Germany).

#### MALDI-TOF–MS analysis

On d_210_, an additional blood sampling was performed on the same individuals selected for blood collection for CBC analysis (n = 24/group). Each additional blood sample was transferred into serum separator tubes with clot activator (2.5 mL volume), left at room temperature for ~ 2 h and then centrifuged at 2500 × g for 10 min. The serum was then aliquoted and stored at −80 °C. At the time of analysis, serum was thawed and diluted to 1:40 in TFA 0.1% solution, desalted and concentrated with C4 zip-tip. The serum was then mixed with a matrix composed of sinapinic acid in acetonitrile/TFA solution. An aliquot (1 µl) of each sample was spotted onto an MSP 96 target ground steel BC (Bruker Daltonics) previously coated with a saturated solution of sinapinic acid in 100% ethanol. Four independent spectra (500 shots, each in a random position) were analysed for each sample using a MALDI-TOF Microflex LRF mass spectrometer equipped with FlexControl software (version 3.4, Bruker Daltonics).

All spectra were recorded in the positive ion linear mode within the mass range 4000–20,000 Da using cytochrome c as the internal standard. Differential peak analysis was performed using ClinProTools software (version 3.0, Bruker Daltonics), with baseline subtraction, total average spectra calculation at a resolution of 800, and data smoothing performed by the Savitzky-Golay algorithm.

#### Statistical analysis

Per each parameter, outlier analysis was conducted applying either the interquartile range (IQR) rule, thus eliminating values above Q3 + 1.5*IQR and those below Q1-1.5*IQR, or the standard deviation (SD) rule, thus eliminating values above the mean + 3SD and below the mean-3SD, depending on the normality of the dataset being analysed. For the TPC analysis, no outlier analysis was conducted due to the limited dataset available.

TPC feed analysis and performance parameters (BW, ADG, FCR, AIDI_H,S,F_) were analysed for t1 and t2 separately. Analysis on haematological parameters were conducted separately on samples collected at d_0_ (n = 80) and at d_210_ (n = 48). An additional analysis was conducted on the percentage variation for each parameter for the animals whose blood had been sampled in both time frames (n = 48). Per each blood parameter, the percentage variation was calculated as follows:

$$\frac{({d}_{210}-{d}_{0})}{{d}_{0}}\times 100$$

Per each parameter, the normality of the data distribution for each treatment (CTR/T) was assessed using the Shapiro–Wilk test. If both datasets presented a normal distribution, the homogeneity of variances was checked using F tests. If both criteria were met, a two-sample t-test was performed; whereas if one or both datasets presented a non-normal distribution, the Wilcoxon rank-sum test was performed.

Animal age at d_0_, d_150_ and d_210_, as well as performance parameters (BW, ADG, FCR, AIDI_H,S,F_), were analysed at the replicate level to identify potential differences using the ANOVA model if sphericity (Mauchly’s Test), normality (Shapiro–Wilk test) and homogeneity of variances (Levene test) were present. If the criteria were not met, a Kruskal Wallis test was conducted, with the Dunn’s test as post-hoc.

A correlation analysis was conducted between AIDI_H,S,F_ and the THI values recorded at the time of data collection. Additionally, linear models including an interaction term (THI × Group) were constructed to evaluate whether the effect of THI on AIDI_H,S,F_ varied significantly between treatments.

All statistical analyses were conducted in R studio (version 4.3.2). Significance was assumed for p-values < 0.05.

## Results

### Oxidative status of the experimental diets

Total phenolic content (Table 3) of the T feed was higher (p < 0.001) than in CTR feed during both phases, while TBARS analysis revealed no differences in lipid oxidation between the two groups at either time point (Table 4).

**Table 3:** TPC of experimental feeds administered to beef cattle during the fattening and finishing phases.

TPC, mg_GAE_/g_feed_			
Time	(Mean ± SD)_CTR_	(Mean ± SD)_T_	p-value
t1	5.71 ± 1.62	14.98 ± 1.87	p < 0.001
t2	5.11 ± 0.54	10.54 ± 1.42	p < 0.001

TPC – total phenolic content; CTR – control group; T – test group; t1- fattening phase; t2 – finishing phase; SD – standard deviation; p-value threshold for significance = 0.05.

**Table 4:** Lipid oxidation assessed through TBARS assay of experimental feeds administered to beef cattle during the fattening and finishing phases.

TBARS, µmol_MDA_/g_feed_			
Time	(Mean ± SD)_CTR_	(Mean ± SD)_T_	p-value
t1	0.31 ± 0.10	0.34 ± 0.05	0.214
t2	0.26 ± 0.05	0.32 ± 0.02	0.200

TBARS – thiobarbituric acid reactive substances; MDA – malondialdehyde; CTR – control group; T – test group; t1 – fattening phase; t2 – finishing phase; SD – standard deviation; p-value threshold for significance = 0.05.

### Growth performances

The inclusion of HS in the diet did not result in significant differences in BW or ADG between CTR and T at any time point, while differences between animals from the T and CTR groups were found for AIDI_F_ and AIDI_S_ (Table 5). Specifically, AIDI_F_ was higher in the T groups during both t1 (p = 0.007) and t2 (p = 0.044), while AIDI_S_ was lower at t1 (p = 0.002). The FCR was higher in T during the fattening phase (p = 0.023).

**Table 5:** Average individual BW, ADG, AIDI_H,S,F_ and FCR of beef bulls in the control and test groups.

	(Mean ± SD)_CTR_	(Mean ± SD)_T_	p-value
BW, kg			
d_0_	282.00 ± 66.27	281.38 ± 64.09	0.966
d_150_	513.26 ± 51.72	513.42 ± 62.07	0.990
d_210_	619.72 ± 56.84	616.76 ± 56.98	0.454
ADG, kg/day			
t1	1.330 ± 0.241	1.358 ± 0.251	0.626
t2	1.196 ± 0.383	1.191 ± 0.362	0.958
overall	1.293 ± 0.233	1.282 ± 0.246	0.857
AIDI_H_, kg			
t1	0.915 ± 0.357	0.838 ± 0.371	0.353
t2	1.203 ± 0.304	1.086 ± 0.240	0.227
AIDI_S_, kg			
t1	0.473 ± 0.220	0.469 ± 0.234	0.943
t2	0.908 ± 0.266	0.637 ± 0.200	0.002
AIDI_F_, kg			
t1	9.449 ± 1.675	10.478 ± 1.632	0.007
t2	10.150 ± 2.308	11.882 ± 2.416	0.044
FCR, kg/kg			
t1	7.073 ± 1.158	7.647 ± 1.312	0.054
t2	8.860 ± 2.386	10.666 ± 3.374	0.023
overall	7.752 ± 1.611	8.356 ± 1.416	0.049

BW – body weight; ADG – average daily gain; AIDI_H_ – average individual daily hay intake; AIDI_S_ – average individual daily straw intake; AIDI_F_ – average individual daily feed intake; FCR – feed conversion ratio; CTR – control group; T – test group; d_0_—trial start day; d_150_ – diet shift day (fattening to finishing); d_210_ – slaughter day; t1 – fattening phase; t2 – finishing phase; overall – total trial period; SD – standard deviation; p-value threshold for significance = 0.05.

Comparisons between replicates in terms of growth performance parameters (BW, ADG, AIDI_H,S,F_, FCR) revealed differences between replicates of the same pair (CTR1 vs. T1; CTR2 vs. T2; CTR3 vs. T3; CTR4 vs. T4) of the same treatment group (CTR1 vs CTR 2 vs CTR3 vs CTR4 and T1 vs T2 vs T3 vs T4), as well as between different pairs and treatments (CTR1 vs CTR 2 vs CTR3 vs CTR4 vs T1 vs T2 vs T3 vs T4). The significant results of these comparisons are reported in S5, Supplementary Material.

Correlation analysis (Table 6) between THI and AIDI_H,S,F_ revealed a significant negative correlation for AIDI_S_ in the CTR group only (p < 0.001) (see S8 to S10, Supplementary Material). The linear model results (Table 7) confirmed a significant general effect of THI on AIDI_S_, although the interaction with the treatment group (CTR/T) was not significant (p = 0.078). No significant THI effects were found for AIDI_H_ and AIDI_F_.

**Table 6:** Correlations of THI x AIDI_H,S,F_ for beef bulls in the control and test groups.

AIDI_H,S,F_ xTHI	CTR	T	p-value_CTR_	p-value_T_
AIDI_H_	−0.14	−0.03	0.34	0.86
AIDI_S_	−0.56	−0.23	< 0.001	0.11
AIDI_F_	−0.05	−0.13	0.75	0.36

AIDI_H_ – average individual daily hay intake; AIDI_S_ – average individual daily straw intake; AIDI_F_ – average individual daily feed intake; THI – temperature humidity index; CTR – control group; T – test group; p-value threshold for significance = 0.05.

**Table 7:** Interaction model results for THI x AIDI_H,S,F_ for beef bulls in the control and test groups.

Intake variable	Intercept	THI	Group	THI × Group	p-value_THI_	p-value_Group_	p-value_THI×Group_
AIDI_H_	1.11	< −0.01	−0.25	< 0.01	0.682	0.488	0.689
AIDI_S_	1.37	−0.01	−0.50	0.01	< 0.001	0.043	0.078
AIDI_F_	10.23	−0.01	2.06	−0.01	0.599	0.292	0.684

AIDI_H_ – average individual daily hay intake; AIDI_S_ – average individual daily straw intake; AIDI_F_ – average individual daily feed intake; THI – temperature humidity index; p-value threshold for significance = 0.05.

### Haematological analysis

Analysis of the (%) changes in haematological parameters between d_0_ and d_210_ revealed significant differences between the two groups (Table 8). The reduction in WBC (10^3^ cells/μL) was greater in T than in CTR (p = 0.035). The RDW (%) increased in T but decreased in CTR (p = 0.035). The increase in MPV (fL) was smaller in T than in CTR (p = 0.034). No significant differences were observed for the changes in any of the other haematological parameters.

**Table 8:** Changes in haematological parameters [(d_210_-d_0_)%] for beef bulls (n = 48) in the control and test groups.

Parameter	(Mean ± SD)_CTR_	(Mean ± SD)_T_	p-value
WBC, 10^3^ cells/μL	−22.14 ± 18.78	−32.92 ± 13.42	0.035
RBC, 10^6^ cells/μL	−8.51 ± 9.05	−6.30 ± 11.99	0.523
HGB, g/dL	13.70 ± 11.37	16.32 ± 12.07	0.470
HCT, %	19.96 ± 12.53	22.25 ± 13.85	0.575
MCV, fL	31.01 ± 7.10	30.94 ± 7.26	0.876
MCH, pg	24.44 ± 9.23	24.33 ± 9.82	0.846
MCHC, g/dL	−4.87 ± 5.94	−4.71 ± 4.44	0.916
CHCM, g/dL	−5.31 ± 3.35	−3.50 ± 3.09	0.073
CH, pg	23.58 ± 6.51	26.37 ± 8.36	0.233
RDW, %	−1.96 ± 10.38	4.28 ± 8.31	0.035
HDW, g/dL	−10.59 ± 19.54	−2.82 ± 8.01	0.149
PLT, 10^3^ cells/μL	41.09 ± 63.30	0.10 ± 68.83	0.876
MPV, fL	26.70 ± 27.07	10.51 ± 27.87	0.034
Neut, 10^3^ cells/μL	−7.61 ± 34.57	−24.19 ± 26.88	0.085
Lymph, 10^3^ cells/μL	−28.09 ± 20.03	−36.30 ± 15.52	0.138
Mono, 10^3^ cells/μL	−26.83 ± 64.94	−23.56 ± 49.53	0.534
Eos, 10^3^ cells/μL	79.61 ± 13.78	69.59 ± 52.73	0.519
Baso, 10^3^ cells/μL	−32.98 ± 66.53	−51.34 ± 28.78	0.503
LUC, 10^3^ cells/μL	Inf	−24.20 ± 58.60	0.181

WBC – white blood cells; RBC – red blood cells; HGB – haemoglobin; HCT (%) – haematocrit (percentage); MCV – mean corpuscular volume; MCH – mean corpuscular haemoglobin; MCHC – mean corpuscular haemoglobin concentration; CHCM – Cellular Haemoglobin Concentration Mean; CH – Cellular Haemoglobin; RDW (%) – Red Cell Distribution Width (percentage); HDW – Haemoglobin Distribution Width; PLT – Platelet Count; MPV – mean platelet volume; Neut – neutrophils; Lymph – lymphocytes; Mono – monocytes; Eos – eosinophils; Baso – basophils; LUC – large unstained cells; CTR – control group; T – test group; SD – standard deviation; p-value threshold for significance = 0.05.

The comparison of haematological parameter mean values between the CTR and T groups at d_0_ and d_210_ are provided in S6, Supplementary Material.

### MALDI-TOF analysis

The MALDI-TOF results are reported in S7, Supplementary material. The serum protein profile reported stands within the 4000–20,000 m/z range. A total of 112 peaks were detected, and no significant differences were observed between the groups for any of the serum profile peaks within the range considered.

## Discussion

The innovative use of the agro-industrial by-product proposed in the present study aligns with contemporary agricultural practices aiming to prioritize sustainability and resource efficiency^36^. Through the employment of HS as a feed ingredient in beef cattle nutrition, our study aimed to verify the possibility of repurposing this by-product from the food supply chain, currently classified as waste, as supported by research demonstrating its antioxidant and antimicrobial properties^37,38^. To this end, HS inclusion in the test diets was performed alongside a reduction in the feed’s corn and barley content^39,40^.

The T feeds used in both phases (fattening and finishing) were particularly enriched in terms of EE and fibre (namely NDF and ADF), showing higher mean values than the CTR feeds. The compositional differences can be attributed to the dietary inclusion of HS, in line with the chemical composition of the latter (S2, Supplementary Material) and previous findings^11,41^. This outcome is important to discuss in terms of the feed intake results and emphasizes how HS significantly enhanced the feed’s nutritional matrix, even with a modest inclusion rate (8% of total feed composition), thus underlining the by-product’s potential technological and functional values in cattle diets.

Focusing on the oxidative status, the T feeds used in both phases contained higher levels of polyphenols (Table 3) – bio-active compounds with a well-documented antioxidant effect^42–44^. Contrary to other findings in the literature, this enrichment was not mirrored in the TBARS assay results (Table 4), which did not detect any differences between groups in terms of the oxidation levels of the feeds’ lipidic fraction^45^. Nonetheless, this outcome was not unexpected since the feed concentrates were freshly prepared on the farm every two weeks, thus stored for relatively short periods (less than 2 weeks on average), with the deliberate intention of providing the animals with the freshest product possible. More importantly, this finding implies that a significant fraction of the antioxidant potential of HS polyphenols may have been directly transferred to the animals through the diet. These findings position HS as a promising tool to enhance the stability and nutritional value of cattle diets, which is particularly relevant given the central role of lipid oxidation in limiting feed quality and shelf-life within the livestock sector^46,47^.

With regard to animal performances, no differences were found between the two groups in terms of BW or ADG. However, HS inclusion modulated the feed and straw intake patterns. Individuals of the T group presented lower AIDI_S_ at t2 and when considering the whole trial period and greater AIDI_F_ for the whole trial period than those belonging to CTR, ultimately leading to a less favourable FCR for the T group (Table 5). Various considerations can be made from these results. First, the HS inclusion did not negatively impact feed palatability. However, as HS have been characterized for their high content of tannins^48^, widely known for their astringent effect which can negatively impact feed intake^49^, we had expected to see a possible negative effect of HS inclusion on palatability. Instead, our finding aligns with those from previous research which showed that tannin supplementation below 2% of dietary DM does not affect feeding behaviour or ADG^50^. Given that the TPC values measured in the feeds of both trial phases (t1 and t2, see Table 3) were below this threshold, it is reasonable to deduce that the tannin levels were also within a safe range, explaining the absence of any negative effect on AIDI_F_. Moreover, considering that the HS employed were collected after industrial roasting in confectionary plants, it is possible that the thermic treatment could have contributed to lowering the antinutritional factors naturally present in raw HS^51^.

Second, the different feeding behaviours exhibited by the two groups, with animals of CTR group consuming greater quantities of straw and lower amounts of feed than those in the T group, can be attributed to the HS chemical profile, specifically to their notable fibre fraction. In fact, one of the factors affecting feed intake in ruminants is the physical limitation of rumen fill, directly influenced by the amount of NDF consumed relative to body weight^52^. Animals in the T group, having ingested larger quantities of feed with a higher NDF content than CTR feed, were consequently less inclined to consume the forage portion of the ration.

These results highlight a potential strategic role of HS in beef cattle nutrition. The sector is, in fact, characterized by feeding strategies primarily based on concentrate feeds, often at the expense of the forage fraction. While the primary aim of such plans would be to maximize growth performance, they also predispose the animals to metabolic disorders such as ruminal acidosis^53,54^. In this context, the capacity of HS to enrich the feed with structural fibre would not only constitute a nutritional adjustment but it would also represent a novel solution to mitigate one of the sector’s most costly and persistent issues. Further research should, therefore, assess whether the increased fibre content resulting from HS inclusion could make a significant contribution to reducing the risk of acidosis and related metabolic disorders in beef cattle.

Third, the higher fibre content of the T feed might have also impacted the FCR. Animals in the T group required more feed per unit of weight gain compared with those in the CTR group during the t2 phase (p = 0.023), which likely influenced the FCR calculated for the entire trial (p = 0.049) (Table 5). These findings are consistent with previous research on lambs fed with HS-enriched diets^55^.

Despite the iso-energetic formulations of the T and CTR diets, the higher fibre content of the T feed likely resulted in a lower energy density per volume unit. Hence, despite providing the same amount of energy per unit of weight, the T feed had a different energy:volume ratio, explaining the greater AIDI_F_ compared with CTR feed. This result, considered in parallel with the equivalent BW values recorded for both groups, ultimately led to a higher FCR in the T group. The discrepancy between the potential benefits of the T feed on the ruminal environment and the less favourable FCR observed in the T group underscores a key limitation of using FCR as a sole indicator of production efficiency, as it does not account for the nutritional quality of the feed, nor for the human-edible portion or value of the resulting product^56,57^. Therefore, although the difference in FCR between the two groups is significant, its practical relevance in the context of beef production should be interpreted with caution. To determine whether this variation truly reflects a less efficient production model, broader evaluations are necessary, such as economic and sustainability assessments, as well as aspects related to market positioning and consumers’ sensory and perceptual responses to the product^58,59^.

Finally, performance differences between the two experimental groups may also be linked to variations in the ruminal microbiome. Paz et al.^60^ reported that up to 20% of the variation in production and efficiency traits in beef cattle (e.g., AIDI_F_, ADG and FCR) can be attributed to microbiome differences^61^. Not only have HS been reported to present in vitro antimicrobial properties, but one of the product’s main classes of bioactive compounds, tannins, is receiving growing attention from the scientific community due to its potential role in modulating the ruminal microbiome, particularly with respect to methanogenic bacteria^62^. Based on these findings, future studies should explore the impact of HS inclusion on the composition and functionality of the rumen microbiota, as well as its potential effects on methane emissions.

Regarding the animals’ health status, haematological parameters recorded at both time points were consistent with a physiological status^63^. Of the most relevant differences detected between the two groups (Table 8), the T group showed a more pronounced reduction in WBC count (p = 0.035) as result of a cumulative decrease in both neutrophils and lymphocytes. Although these reductions were not significant when assessed individually (S6, Supplementary Material), their combined effect may have driven the overall variation in WBC. The greater reduction in WBC in the T group may be linked to the HS phenolic content, the antioxidant properties of which have been reported to reduce oxidative stress and modulate immune cell recruitment^64–66^. Given that all values remained within physiological ranges, this effect could reflect an improved systemic homeostasis rather than an impairment of immune function. In parallel, MPV values presented a greater increase in the CTR group than in the T group. Since MPV reflects platelet activation and reactivity, this pattern suggests that animals fed HS had a lower systemic level of platelet activation which, according to literature, could be attributed to their phenolic content^67^. Future analyses should assess the functional activity of neutrophils as well as quantify them to understand the immunological implications of such shifts better. The combined WBC and MPV findings suggest a possible modulatory role of HS bioactive compounds on inflammatory and oxidative processes, which require further targeted investigation. Concerning RDW (%), despite the differences detected at d_0_, both T and CTR groups showed similar erythrocyte profiles by d_210_, indicating a convergence in red blood cell characteristics over time^68^.

These results, together with the MALDI-TOF evaluations, suggest that dietary inclusion of HS does not negatively affect the animals’ blood parameters, nor does it appear to introduce any major alterations in the serum proteomic profile, at least within the analytical m/z range considered in the present study.

Potential limitations of the study could be attributed to the HS inclusion level. The value (8%) was chosen following the “principle of precaution”, due to the lack of literature on the matter. However, on the basis of our results, in future trials it would be advisable to explore the effects of varying HS inclusion levels in cattle diets to better define its optimal application. In this context, it is particularly noteworthy to report the findings of a similar nutritional study by Renna et al.^11^. In their trial, Valdostana Red Pied dairy cows were divided into two dietary groups (CTR and T), with the test group receiving a diet containing twice the HS inclusion level used in our study (16%). Their results showed a reduction in feed intake in the T group compared with the CTR group. Determining the optimal rate of inclusion will require balancing potential drawbacks such as reduced feed intake with the advantages of repurposing a by-product that would otherwise go to waste, thereby enhancing circularity in the sector.

A major limitation of this study can be attributed to the differences among replicates in terms of age and BW at d_0_ (see S4 and S5, Supplementary Material). Additionally, the staggered entries of the replicate pairs into the trial at four different times of the year could have played a role in the results, as highlighted by the FCR differences among replicates (see S5, Supplementary material). Nonetheless, it should be considered that the heterogeneity observed in the study sample is primarily due to the commercial nature of the setup where the trial was conducted, which needed to accommodate economic and management necessities. On the other hand, the commercial nature of the environment where the trial was conducted allowed us to explore the effects of the HS inclusion in a real-world scenario. This characteristic should also, therefore, be considered a strength of the study. The long duration of the trial is also a strength, being (to the best of the authors’ knowledge) one of the longest involving beef cattle reported in the current literature in the field of cattle diet sustainability.

## Conclusions

This study aimed to assess the implications of HS inclusion in beef cattle diets in view of the principles of circular economy. A key result was the significant boost in the TPC levels of the T diets, reporting values nearly three times greater during the fattening phase and twice as high during the finishing phase compared with CTR diets. This enhancement could present potential positive implications for the feed’s nutritional profile and should be the focus of future investigations, both in animal and human nutrition.

At the same time, our results indicate that incorporating hazelnut skins into ruminant diets did not compromise growth performance or overall health. Hence, the present study supports the viability of using hazelnut skins as a circular feed ingredient with the scope of improving the sustainability of the food supply chain.

## Supplementary Information

Supplementary Information.

## Data Availability

The analysed data from this experiment are available upon request from the corresponding author.

## Abbreviations

ADF: Acid detergent fibre
ADG: Average daily gain
ADL: Acid detergent lignin
AIDI_F_: Average individual daily intake of feed
AIDI_H_: Average individual daily intake of hay
AIDI_S_: Average individual daily intake of straw
BW: Body weight
CP: Crude protein
CTR: Control
DM: Dry matter
EE: Ether extract
FCR: Feed conversion ratio
HS: Hazelnut skins
NDF: Neutral detergent fibre
SD: Standard deviation
T: Test
TBARS: Thiobarbituric Acid Reactive Substances
THI: Temperature – humidity index
TPC: Total phenolic content
