# Supplementary material for: Hazelnut skins as a new sustainable ingredient for beef cattle diets
Source: Sci Rep. 2025 Nov 6;15:38926. doi: 10.1038/s41598-025-22896-1 (PMC12592447; doi:10.1038/s41598-025-22896-1)
Supplement: Supplementary file 1 — Supplementary Information. [file 41598_2025_22896_MOESM1_ESM.doc]

Supplementary Material

S1. Ingredients of experimental feeds administered to beef cattle during fattening and finishing phases.

| Ingredients | Fattening feed | | | Finishing feed | |
| --- | --- | --- | --- | --- | --- |
|  | CTR (%) | T (%) | CTR (%) | | T (%) |
| Coarse corn meal | 63.6 | 56.2 | 68.9 | | 60.9 |
| Soybean meal | 4.5 | 4.5 | 11.7 | | 11.7 |
| Sunflower meal | 5.5 | 5.5 | 0.0 | | 0.0 |
| Beet pulp | 4.9 | 4.9 | 5.9 | | 5.9 |
| Bran | 5.3 | 5.3 | 5.0 | | 5.0 |
| Soybean oil | 0.0 | 0.0 | 1.0 | | 1.0 |
| Wheat distiller | 5.2 | 5.2 | 0.0 | | 0.0 |
| Barley | 5.5 | 4.9 | 0.0 | | 0.0 |
| Extruded flaxseeds | 0.3 | 0.3 | 1.0 | | 1.0 |
| Hydrogenated fat | 0.3 | 0.3 | 0.5 | | 0.5 |
| Coalvitor Proteo^a^ | 2.5 | 2.5 | 0 | | 0.0 |
| Coalvitor CL^b^ | 0.0 | 0.0 | 3.5 | | 3.5 |
| Tamp-one^c^ | 0.5 | 0.5 | 0.5 | | 0.5 |
| Multi acid dry^d^ | 0.9 | 0.9 | 0.9 | | 0.9 |
| Urofast^e^ | 1.0 | 1.0 | 1.0 | | 1.0 |
| HS | 0.0 | 8.0 | 0 | | 8.0 |

*^a^ingredients: calcium carbonate from ground lime rocks, sodium bicarbonate, magnesium oxide, sodium chloride, monobicalcium phosphate, common wheat tritium, sulphur flower, dried yeast (Saccharomyces Cerevisiae); analytical components %: ash insoluble in HCL 3.12, calcium 16.5, phosphorus 0.57, magnesium 3.03, sodium 6.56; ^b^ingredients: calcium carbonate from ground lime rocks, monobicalcium phosphate, common wheat tritium, sodium bicarbonate, magnesium oxide, sodium chloride, sulphur flower, dried yeast (Saccharomyces Cerevisiae); analytical components %: ash insoluble in HCL 3.97, calcium 19.8, phosphorus 2.73, magnesium 3.49, sodium 4.19; ^c^ingredients: sodium bicarbonate, magnesium oxide, calcium carbonate from ground lime rocks, common wheat tritum; analytical components %: humidity 11.44, calcium 5.7, phosphorus 0.01, magnesium 10.07, sodium 17.80; ^d^ingredients: wheat flour, yeast inactivated by Saccharomyces cerevisiaea; analytical components %: moisture 10.0, crude protein 12.5, crude fibre 2.0, ash 2.5, crude fat 1.5, calcium 0.05, phosphorus 0.15, sodium 0.01, magnesium 0.02; ^e^ingredients: sodium bicarbonate, calcium carbonate from ground lime rocks, magnesium oxide, common wheat tritium; analytical components %: calcium 10.4, phosphorus 0.06, magnesium 4.65, sodium 12.61; HS- hazelnut skins; CTR – control group; T – test group*

S2. Chemical composition of the hazelnut skins.

| Chemical composition, %^a^ | HS |
| --- | --- |
| DM | 92.19 |
| Ash | 2.17 |
| CP | 6.95 |
| EE | 25.03 |
| NDF | 22.54 |
| ADF | 17.07 |
| ADL | 6.71 |

*HS – hazelnut skin; DM – dry matter; CP – crude protein; EE – ether extract; NDL – neutral detergent fibre; ADF – acid detergent fibre; ADL – acid detergent lignin; ^a^as fresh matter basis*

S3. Chemical composition of experimental feeds administered to beef cattle during fattening and finishing phases.

| Chemical composition, %^a^ | t1 | | t2 | |
| --- | --- | --- | --- | --- |
|  | CTR | T | CTR | T |
| DM | 89.82 | 90.08 | 90.02 | 90.42 |
| Ash | 5.59 | 5.81 | 6.77 | 6.42 |
| CP | 13.65 | 14.11 | 13.78 | 12.82 |
| EE | 3.30 | 4.82 | 3.86 | 5.60 |
| NDF | 15.09 | 16.52 | 12.12 | 12.45 |
| ADF | 5.39 | 6.65 | 4.21 | 4.72 |
| ADL | 0.54 | 1.28 | 0.22 | 0.45 |

*DM – dry matter; CP – crude protein; EE – ether extract; NDL – neutral detergent fibre; ADF – acid detergent fibre; ADL – acid detergent lignin; CTR – control group; T – test group; t1 – fattening phase; t2 – finishing phase; ^a^as fresh matter basis*

S4. Replicates’ age (in days) comparison at d0, d1, d2. Only significant results are reported.

| Time | Repl. 1 | (Mean±SD)_1_ | Repl. 2 | (Mean±SD)_2_ | p-value |
| --- | --- | --- | --- | --- | --- |
| d_0_ | CTR1 | 260.10 ± 27.81 | CTR2 | 208.40 ± 29.87 | 0.01 |
|  | CTR1 | 260.10 ± 27.81 | T2 | 214.50 ± 22.11 | 0.04 |
|  | CTR1 | 260.10 ± 27.81 | CTR4 | 165.30 ± 34.38 | <0.001 |
|  | CTR1 | 260.10 ± 27.81 | T4 | 191.70 ± 36.20 | <0.001 |
|  | T1 | 287.80 ± 45.51 | CTR2 | 208.40 ± 29.87 | <0.001 |
|  | T1 | 287.80 ± 45.51 | T2 | 214.50 ± 22.11 | <0.001 |
|  | T1 | 287.80 ± 45.51 | CTR3 | 232.10 ± 30.92 | <0.001 |
|  | T1 | 287.80 ± 45.51 | T3 | 239.90 ± 21.14 | 0.03 |
|  | T1 | 287.80 ± 45.51 | CTR4 | 165.30 ± 34.38 | <0.001 |
|  | T1 | 287.80 ± 45.51 | T4 | 191.70 ± 36.20 | <0.001 |
|  | T2 | 214.50 ± 22.11 | CTR4 | 165.30 ± 34.38 | 0.02 |
|  | CTR3 | 232.10 ± 30.92 | CTR4 | 165.30 ± 34.38 | <0.001 |
|  | T3 | 239.90 ± 21.14 | CTR4 | 165.30 ± 34.38 | <0.001 |
|  | T3 | 239.90 ± 21.14 | T4 | 191.70 ± 36.20 | 0.02 |
| d_150_ | CTR2 | 384.40 ± 29.87 | CTR1 | 441.10 ± 27.81 | 0.003 |
|  | T2 | 390.50 ± 22.11 | CTR1 | 441.10 ± 27.81 | 0.01 |
|  | CTR3 | 385.10 ± 30.92 | CTR1 | 441.10 ± 27.81 | 0.004 |
|  | T3 | 390.25 ± 21.97 | CTR1 | 441.10 ± 27.81 | 0.03 |
|  | CTR4 | 345.78 ± 29.28 | CTR1 | 441.10 ± 27.81 | <0.001 |
|  | T4 | 365.00 ± 38.32 | CTR1 | 441.10 ± 27.81 | <0.001 |
|  | CTR2 | 384.40 ± 29.87 | T1 | 468.80 ± 45.51 | <0.001 |
|  | T2 | 390.50 ± 22.11 | T1 | 468.80 ± 45.51 | <0.001 |
|  | CTR3 | 385.10 ± 30.92 | T1 | 468.80 ± 45.51 | <0.001 |
|  | T3 | 390.25 ± 21.97 | T1 | 468.80 ± 45.51 | <0.001 |
|  | CTR4 | 345.78 ± 29.28 | T1 | 468.80 ± 45.51 | <0.001 |
|  | T4 | 365.00 ± 38.32 | T1 | 468.80 ± 45.51 | <0.001 |
| d_210_ | CTR1 | 513.30 ± 47.69 | CTR4 | 439.00 ± 20.07 | 0.03 |
|  | T1 | 546.60 ± 54.86 | CTR3 | 469.60 ± 27.29 | 0.03 |
|  | T1 | 546.60 ± 54.86 | CTR4 | 439.00 ± 20.07 | <0.001 |

*CTR – control group; T – test group; d_0_ – trial start day; d_150_ – diet shift day (fattening to finishing); d_210_ – slaughter day; SD – standard deviation; p-value threshold for significance = 0.05*

S5. BW, ADG, AIDI_H,S,F_, FCR comparisons among Control and Test replicates. Only significant results are reported.

| Time | Repl. 1 | (Mean±SD)_1_ | Repl. 2 | (Mean±SD)_2_ | p-value |
| --- | --- | --- | --- | --- | --- |
| BW, kg | | | | | |
| d_0_ | CTR1 | 341.00±46.30 | CTR2 | 267.00±54.40 | <0.001 |
|  | CTR1 | 341.00±46.30 | CTR4 | 231.00±55.80 | <0.001 |
|  | CTR1 | 341.00±46.30 | T2 | 268.00±47.00 | 0.0129 |
|  | CTR1 | 341.00±46.30 | T4 | 233.00±51.90 | <0.001 |
|  | CTR3 | 290.00±61.30 | T4 | 233.00±51.90 | 0.0433 |
|  | T1 | 336.00±35.30 | CTR2 | 267.00±54.40 | <0.001 |
| D_150_ | CTR1 | 551.00±48.00 | CTR2 | 502.00±40.60 | 0.0206 |
|  | CTR1 | 551.00±48.00 | CTR3 | 486.00±45.20 | <0.001 |
|  | CTR1 | 551.00±48.00 | T2 | 501.00±38.10 | 0.0292 |
|  | CTR1 | 551.00±48.00 | T4 | 491.00±71.90 | 0.0105 |
|  | T1 | 567.00±47.20 | CTR2 | 502.00±40.60 | 0.0045 |
|  | T1 | 567.00±47.20 | T2 | 501.00±38.10 | 0.0071 |
| d_210_ | No differences | | | | 0.3532 |
| ADG, kg/day | | | | | |
| t1 | CTR4 | 1.56±0.166 | CTR1 | 1.16±0.211 | <0.001 |
|  | CTR4 | 1.56±0.166 | CTR2 | 1.34±0.210 | 0.043 |
|  | CTR4 | 1.56±0.166 | CTR3 | 1.28±0.216 | 0.011 |
|  | CTR4 | 1.56±0.166 | T1 | 1.27±0.267 | 0.008 |
|  | CTR4 | 1.56±0.166 | T3 | 1.33±0.310 | 0.024 |
|  | T4 | 1.46±0.254 | CTR1 | 1.16±0.211 | 0.013 |
| t2 | No differences | | | | 0.125 |
| overall | No differences | | | | 0.079 |
| AIDI_H_, kg | | | | | |
| t1 | CTR1 | 1.06±0.37 | CTR4 | 0.699±0.23 | 0.023 |
|  | CTR1 | 1.06±0.37 | T3 | 0.619±0.28 | 0.011 |
|  | CTR1 | 1.06±0.37 | T4 | 0.698±0.27 | 0.026 |
|  | CTR2 | 1.02±0.29 | CTR4 | 0.699±0.23 | 0.044 |
|  | CTR2 | 1.02±0.29 | T3 | 0.619±0.28 | 0.021 |
| t2 | No differences | | | | 0.536 |
| AIDI_S_, kg | | | | | |
| t1 | No differences | | | | 0.800 |
| t2 | No differences | | | | 0.092 |
| AIDI_F_, kg | | | | | |
| t1 | T1 | 11.5±1.06 | CTR1 | 9.96±1.01 | 0.030 |
|  | T1 | 11.5±1.06 | CTR2 | 9.03±1.99 | 0.001 |
|  | T1 | 11.5±1.06 | CTR3 | 8.84±1.65 | <0.001 |
|  | T1 | 11.5±1.06 | CTR4 | 9.81±1.95 | 0.026 |
|  | T1 | 11.5±1.06 | T3 | 9.66±0.93 | 0.019 |
|  | T1 | 11.5±1.06 | T4 | 9.69±1.20 | 0.015 |
| t2 | No differences | | | | 0.129 |
| FCR, kg/kg | | | | | |
| t1 | CTR1 | 8.87±1.88 | CTR2 | 6.92±1.15 | 0.012 |
|  | CTR1 | 8.87±1.88 | CTR3 | 7.07±1.25 | 0.021 |
|  | CTR1 | 8.87±1.88 | CTR4 | 6.35±0.60 | <0.001 |
|  | CTR1 | 8.87±1.88 | T4 | 6.81±1.23 | 0.010 |
|  | T1 | 9.41±2.10 | CTR2 | 6.92±1.15 | 0.002 |
|  | T1 | 9.41±2.10 | CTR3 | 7.07±1.25 | 0.005 |
|  | T1 | 9.41±2.10 | CTR4 | 6.35±0.60 | <0.001 |
|  | T1 | 9.41±2.10 | T4 | 6.81±1.23 | 0.002 |
|  | T2 | 7.77±0.84 | CTR4 | 6.35±0.60 | 0.016 |
| t2 | T1 | 14.3±7.44 | CTR2 | 8.72±3.20 | 0.018 |
|  | T1 | 14.3±7.44 | CTR4 | 10.1±5.79 | 0.025 |
|  | T1 | 14.3±7.44 | T4 | 7.97±1.14 | 0.005 |
|  | T2 | 11.8±3.82 | CTR2 | 8.72±3.20 | 0.043 |
|  | T2 | 11.8±3.82 | T4 | 7.97±1.14 | 0.014 |
|  | T3 | 11.7±3.11 | CTR2 | 8.72±3.20 | 0.027 |
|  | T3 | 11.7±3.11 | CTR4 | 10.1±5.79 | 0.035 |
|  | T3 | 11.7±3.11 | T4 | 7.97±1.14 | 0.008 |
| overall | CTR1 | 8.74±1.30 | CTR2 | 7.23±1.10 | 0.023 |
|  | CTR1 | 8.74±1.30 | CTR4 | 7.05±1.33 | 0.016 |
|  | CTR1 | 8.74±1.30 | T4 | 7.30±1.08 | 0.043 |
|  | T1 | 10.2±2.66 | CTR2 | 7.23±1.10 | 0.003 |
|  | T1 | 10.2±2.66 | CTR3 | 7.89±2.18 | 0.024 |
|  | T1 | 10.2±2.66 | CTR4 | 7.05±1.33 | 0.002 |
|  | T1 | 10.2±2.66 | T4 | 7.30±1.08 | 0.007 |
|  | T2 | 9.29±2.25 | CTR2 | 7.23±1.10 | 0.012 |
|  | T2 | 9.29±2.25 | CTR4 | 7.05±1.33 | 0.008 |
|  | T2 | 9.29±2.25 | T4 | 7.30±1.08 | 0.024 |
|  | T3 | 8.55±1.17 | CTR2 | 7.23±1.10 | 0.039 |
|  | T3 | 8.55±1.17 | CTR4 | 7.05±1.33 | 0.026 |

*BW – body weight; AGD – Average Daily Gain; AIDI_H_ – average individual daily hay intake; AIDI_S_ – average individual daily straw intake; AIDI_F_– average individual daily feed intake; FCR – Feed Conversion Ratio; CTR – control group; T – test group; d_0_  – trial start day; d_150_ – diet shift day (fattening to finishing); d_210_ – slaughter day; t1 – fattening phase; t2 – finishing phase; overall – total trial period; SD – standard deviation;* *p-value threshold for significance = 0.05*

S6. Haematological parameters (d0; d2) of beef bulls (n=80; n=48) in Control and Test groups.

| Parameter | (Mean±SD)_CTR_ | (Mean±SD)_T_ | p-value |
| --- | --- | --- | --- |
| WBC, 10^3^ cells/μL | | | |
| d_0_ | 9.06±0.29 | 8.92±0.28 | 0.786 |
| d_210_ | 7.10 ±0.32 | 6.27±0.23 | 0.038 |
| RBC, 10^6^ cells/μL | | | |
| d_0_ | 9.86±0.18 | 10.15±0.16 | 0.247 |
| d_210_ | 9.05±0.18 | 9.48±0.21 | 0.129 |
| HGB, g/dL | | | |
| d_0_ | 12.22±0.16 | 12.12±0.17 | 0.99 |
| d_210_ | 13.68±0.20 | 13.98±0.22 | 0.32 |
| HCT, % | | | |
| d_0_ | 32.46±2.87 | 31.69±2.79 | 0.238 |
| d_210_ | 38.07±0.56 | 38.40±0.67 | 0.744 |
| MCV, fL | | | |
| d_0_ | 32.78±0.51 | 31.35±0.40 | 0.031 |
| d_210_ | 42.26±0.69 | 40.74±0.68 | 0.123 |
| MCH, pg | | | |
| d_0_ | 12.38±0.19 | 11.98±0.14 | 0.088 |
| d_210_ | 15.20±0.29 | 14.78±0.29 | 0.302 |
| MCHC, g/dL | | | |
| d_0_ | 37.79±0.28 | 37.89±0.10 | 0.146 |
| d_210_ | 35.98±0.32 | 36.44±0.27 | 0.205 |
| CHCM, g/dL | | | |
| d_0_ | 37.22±0.18 | 37.43±0.16 | 0.386 |
| d_210_ | 35.12±0.25 | 36.06±0.21 | 0.006 |
| CH, pg | | | |
| d_0_ | 12.20±0.20 | 11.75±0.17 | 0.0871 |
| d_210_ | 14.81±0.27 | 14.69±0.23 | 0.742 |
| RDW, % | | | |
| d_0_ | 20.05±1.42 | 19.21±1.25 | 0.007 |
| d_210_ | 19.83±0.31 | 19.58±0.25 | 0.543 |
| HDW, g/dL | | | |
| d_0_ | 1.99±0.02 | 1.96±0.02 | 0.146 |
| d_210_ | 1.90±0.04 | 1.94±0.05 | 0.749 |
| PLT, 10^3^ cells/μL | | | |
| d_0_ | 462.32±27.09 | 478.10±29.64 | 0.696 |
| d_210_ | 359.96±25.05 | 403.63±27.87 | 0.253 |
| MPV, fL | | | |
| d_0_ | 6.17±0.07 | 6.36±0.10 | 0.59 |
| d_210_ | 8.13±0.32 | 7.16±0.28 | 0.021 |
| Neut, 10^3^ cells/μL | | | |
| d_0_ | 2.89±0.16 | 2.80±0.15 | 0.621 |
| d_210_ | 2.88±0.27 | 2.15±0.12 | 0.011 |
| Lymph, 10^3^ cells/μL | | | |
| d_0_ | 5.16±0.18 | 5.47±0.19 | 0.483 |
| d_210_ | 3.66±0.18 | 3.62±0.18 | 0.885 |
| Mono, 10^3^ cells/μL | | | |
| d_0_ | 0.26±0.02 | 0.19±0.01 | 0.019 |
| d_210_ | 0.14±0.02 | 0.13±0.01 | 0.665 |
| Eos, 10^3^ cells/μL | | | |
| d_0_ | 0.21±0.02 | 0.22±0.03 | 0.985 |
| d_210_ | 0.33±0.05 | 0.29±0.04 | 0.35 |
| Baso, 10^3^ cells/μL | | | |
| d_0_ | 0.20±0.03 | 0.15±0.01 | 0.074 |
| d_210_ | 0.11±0.03 | 0.07±0.01 | 0.658 |
| LUC, 10^3^ cells/μL | | | |
| d_0_ | 0.04±0.01 | 0.02±0.02 | 0.004 |
| d_210_ | 0.01±0.00 | 0.01±0.00 | 0.804 |

*WBC – White Blood Cells; RBC – Red Blood Cells; HGB – Haemoglobin; HCT (%) – Haematocrit (percentage); MCV – Mean Corpuscular Volume; MCH – Mean Corpuscular Haemoglobin; MCHC – Mean Corpuscular Haemoglobin Concentration; CHCM – Cellular Haemoglobin Concentration Mean; CH – Cellular Haemoglobin; RDW (%) – Red Cell Distribution Width (percentage); HDW – Haemoglobin Distribution Width; PLT – Platelet Count; MPV – Mean Platelet Volume; Neut – Neutrophils; Lymph – Lymphocytes; Mono – Monocytes; Eos – Eosinophils; Baso – Basophils; LUC – Large Unstained Cells; CTR – control group; T – test group*; *d_0_ – trial start day; d_210_ – slaughter day; SD – standard deviation; p-value threshold for significance = 0.05*

S7. MALDI-TOF-MS serum analysis results from beef bulls’ (n=48) of Control and Test groups.

| Index | m/z | ANOVA | Wilcoxon | (Mean±SD)_CTR_ | (Mean±SD)_T_ |
| --- | --- | --- | --- | --- | --- |
| 1 | 4009.74 | 0.052 | 0.248 | 4.55±1.66 | 3.95±1.08 |
| 2 | 4029.82 | 0.235 | 0.681 | 6.04±5.51 | 4.81±4.39 |
| 3 | 4045.77 | 0.926 | 0.948 | 1.82±0.73 | 1.80±0.70 |
| 4 | 4079.68 | 0.926 | 0.863 | 4.63±2.04 | 4.58±2.22 |
| 5 | 4153.87 | 0.074 | 0.242 | 2.37±1.23 | 1.96±0.99 |
| 6 | 4266.00 | 0.083 | 0.611 | 1.57±0.87 | 1.33±0.45 |
| 7 | 4278.65 | 0.071 | 0.248 | 2.78±4.86 | 1.38±0.94 |
| 8 | 4321.77 | 0.688 | 0.946 | 1.39±0.68 | 1.33±0.58 |
| 9 | 4388.80 | 0.010 | 0.216 | 5.37±4.79 | 3.29±2.06 |
| 10 | 4443.12 | 0.044 | 0.093 | 7.84±2.31 | 8.96±2.52 |
| 11 | 4465.80 | 0.119 | 0.271 | 12.94±4.8 | 14.44±4.81 |
| 12 | 4522.17 | 0.060 | 0.149 | 26.38±11.73 | 31.23±11.69 |
| 13 | 4545.44 | 0.180 | 0.338 | 72.75±31.14 | 80.95±28.26 |
| 14 | 4580.54 | 0.097 | 0.242 | 15.38±6.64 | 17.58±6.31 |
| 15 | 4622.76 | 0.655 | 0.248 | 3.25±1.75 | 3.42±1.31 |
| 16 | 4640.29 | 0.400 | 0.681 | 2.86±0.96 | 3.01±0.67 |
| 17 | 4674.41 | 0.889 | 0.248 | 2.31±1.82 | 2.39±1.45 |
| 18 | 4702.60 | 0.851 | 0.449 | 6.75±8.42 | 6.36±5.51 |
| 19 | 4726.03 | 0.298 | 0.256 | 2.09±1.21 | 2.34±1.12 |
| 20 | 4752.20 | 0.235 | 0.291 | 3.78±1.67 | 4.19±1.68 |
| 21 | 4927.11 | 0.482 | 0.634 | 4.87±2.93 | 5.36±3.16 |
| 22 | 4968.70 | 0.655 | 0.682 | 1.39±0.62 | 1.45±0.50 |
| 23 | 5056.39 | 0.192 | 0.382 | 2.44±2.02 | 3.00±2.22 |
| 24 | 5082.56 | 0.433 | 0.468 | 7.48±6.43 | 8.60±6.63 |
| 25 | 5124.89 | 0.235 | 0.248 | 1.25±0.94 | 1.49±0.96 |
| 26 | 5140.59 | 0.291 | 0.271 | 1.57±0.80 | 1.74±0.76 |
| 27 | 5303.40 | 0.541 | 0.661 | 1.32±0.68 | 1.23±0.57 |
| 28 | 5348.38 | 0.674 | 0.611 | 2.71±3.63 | 2.34±3.63 |
| 29 | 5435.77 | 0.973 | 0.993 | 1.61±0.65 | 1.61±0.77 |
| 30 | 5532.45 | 0.851 | 0.956 | 3.61±1.84 | 3.52±1.42 |
| 31 | 5582.56 | 0.533 | 0.363 | 3.05±1.48 | 2.84±1.47 |
| 32 | 5647.49 | 0.655 | 0.598 | 2.07±1.16 | 1.95±1.10 |
| 33 | 5681.41 | 0.327 | 0.783 | 1.12±0.41 | 1.25±0.81 |
| 34 | 5697.31 | 0.159 | 0.260 | 1.42±0.97 | 1.17±0.82 |
| 35 | 5793.99 | 0.103 | 0.464 | 2.70±1.82 | 2.19±1.11 |
| 36 | 5823.60 | 0.313 | 0.712 | 2.15±1.06 | 3.03±5.83 |
| 37 | 5840.27 | 0.049 | 0.118 | 3.72±2.93 | 5.27±4.12 |
| 38 | 6051.76 | 0.113 | 0.242 | 0.89±0.29 | 0.99±0.34 |
| 39 | 6121.84 | 0.656 | 0.765 | 12.02±4.96 | 12.56±5.22 |
| 40 | 6161.87 | 0.533 | 0.611 | 2.59±0.92 | 2.72±0.86 |
| 41 | 6239.88 | 0.113 | 0.248 | 3.46±1.33 | 3.09±0.98 |
| 42 | 6282.22 | 0.074 | 0.271 | 1.44±0.41 | 1.58±0.33 |
| 43 | 6324.67 | 0.194 | 0.350 | 1.31±0.84 | 1.12±0.52 |
| 44 | 6665.05 | 0.817 | 0.786 | 0.79±0.17 | 0.81±0.21 |
| 45 | 6711.03 | 0.074 | 0.260 | 0.86±0.35 | 1.02±0.49 |
| 46 | 6742.17 | 0.106 | 0.271 | 3.27±1.78 | 3.92±2.18 |
| 47 | 6786.36 | 0.775 | 0.449 | 2.41±1.35 | 2.50±1.05 |
| 48 | 6821.47 | 0.106 | 0.260 | 8.33±4.44 | 9.92±5.26 |
| 49 | 6875.18 | 0.655 | 0.712 | 0.78±0.30 | 0.81±0.31 |
| 50 | 6954.99 | 0.926 | 0.681 | 1.29±0.76 | 1.31±0.62 |
| 51 | 6977.60 | 0.817 | 0.576 | 1.08±1.07 | 1.01±0.76 |
| 52 | 7258.93 | 0.956 | 0.948 | 0.80±0.24 | 0.80±0.25 |
| 53 | 7358.26 | 0.283 | 0.382 | 1.27±0.94 | 1.49±1.02 |
| 54 | 7513.04 | 0.433 | 0.600 | 0.62±0.37 | 0.69±0.49 |
| 55 | 7534.97 | 0.655 | 0.872 | 4.02±2.94 | 4.52±5.59 |
| 56 | 7618.68 | 0.010 | 0.034 | 0.83±0.74 | 0.51±0.33 |
| 57 | 7641.89 | 0.125 | 0.242 | 0.96±0.65 | 0.76±0.60 |
| 58 | 7655.82 | 0.182 | 0.242 | 1.07±0.53 | 0.93±0.54 |
| 59 | 7729.49 | 0.015 | 0.449 | 1.63±3.59 | 0.27±0.13 |
| 60 | 7866.57 | 0.010 | 0.004 | 1.48±2.86 | 0.32±0.10 |
| 61 | 7952.26 | 0.049 | 0.470 | 0.69±0.69 | 0.47±0.18 |
| 62 | 8225.14 | 0.103 | 0.712 | 1.60±2.14 | 1.03±1.07 |
| 63 | 8361.52 | 0.002 | 0.025 | 0.58±0.50 | 0.32±0.13 |
| 64 | 8390.56 | 0.298 | 0.362 | 0.48±0.21 | 0.44±0.17 |
| 65 | 8479.82 | 0.005 | 0.034 | 0.97±0.81 | 0.60±0.28 |
| 66 | 8514.25 | 0.797 | 0.470 | 0.98±1.34 | 0.90±0.86 |
| 67 | 8557.57 | 0.091 | 0.449 | 6.14±14.83 | 2.44±1.75 |
| 68 | 8590.02 | 0.113 | 0.025 | 3.26±2.92 | 2.36±2.68 |
| 69 | 8673.25 | 0.074 | 0.149 | 0.81±0.30 | 0.70±0.31 |
| 70 | 8737.18 | 0.351 | 0.210 | 2.03±1.09 | 2.66±4.39 |
| 71 | 8780.40 | 0.011 | 0.236 | 20.02±19.98 | 11.51±8.93 |
| 72 | 8883.16 | 0.573 | 0.643 | 2.91±2.71 | 2.57±2.47 |
| 73 | 8941.54 | 0.246 | 0.971 | 1.85±2.36 | 1.39±1.36 |
| 74 | 8987.70 | 0.049 | 0.537 | 1.37±1.49 | 0.86±0.57 |
| 75 | 9010.43 | 0.074 | 0.681 | 1.46±1.25 | 1.10±0.55 |
| 76 | 9049.99 | 0.074 | 0.958 | 0.87±1.23 | 0.54±0.26 |
| 77 | 9065.02 | 0.074 | 0.981 | 0.87±1.09 | 0.57±0.24 |
| 78 | 9095.56 | 0.074 | 0.682 | 1.74±1.55 | 1.30±0.64 |
| 79 | 9165.89 | 0.159 | 0.682 | 0.71±0.58 | 0.57±0.33 |
| 80 | 9469.35 | 0.049 | 0.712 | 0.15±0.08 | 0.37±0.68 |
| 81 | 9655.71 | 0.354 | 0.981 | 0.31±0.61 | 0.22±0.24 |
| 82 | 9981.53 | 0.605 | 0.475 | 0.41±0.34 | 0.37±0.31 |
| 83 | 10160.35 | 0.074 | 0.162 | 0.22±0.14 | 0.18±0.10 |
| 84 | 10288.50 | 0.926 | 0.863 | 1.14±1.15 | 1.17±1.22 |
| 85 | 10508.41 | 0.074 | 0.253 | 0.21±0.16 | 0.16±0.11 |
| 86 | 10826.32 | 0.074 | 0.095 | 0.41±0.23 | 0.33±0.21 |
| 87 | 11053.57 | 0.074 | 0.248 | 0.74±1.02 | 0.43±0.53 |
| 88 | 11262.59 | 0.002 | 0.006 | 0.25±0.16 | 0.16±0.08 |
| 89 | 11293.07 | 0.246 | 0.598 | 0.38±0.29 | 0.32±0.20 |
| 90 | 11371.02 | 0.655 | 0.712 | 0.15±0.14 | 0.14±0.12 |
| 91 | 11643.15 | 0.246 | 0.948 | 0.94±0.58 | 1.35±2.38 |
| 92 | 12017.47 | 0.246 | 0.634 | 0.17±0.17 | 0.14±0.10 |
| 93 | 12242.49 | 0.935 | 0.971 | 25.35±10.47 | 25.53±11.94 |
| 94 | 12455.10 | 0.906 | 0.948 | 4.28±1.99 | 4.37±2.50 |
| 95 | 12592.87 | 0.923 | 0.948 | 1.73±0.90 | 1.77±1.09 |
| 96 | 12645.63 | 0.246 | 0.681 | 2.41±2.35 | 1.96±1.27 |
| 97 | 12801.08 | 0.926 | 0.981 | 1.16±0.73 | 1.18±0.83 |
| 98 | 12865.78 | 0.925 | 0.917 | 0.71±0.55 | 0.73±0.53 |
| 99 | 13463.04 | 0.083 | 0.242 | 0.14±0.09 | 0.11±0.07 |
| 100 | 13569.13 | 0.159 | 0.444 | 1.45±2.48 | 0.88±1.32 |
| 101 | 13646.42 | 0.092 | 0.444 | 0.27±0.41 | 0.16±0.14 |
| 102 | 13688.61 | 0.067 | 0.242 | 0.41±0.52 | 0.25±0.25 |
| 103 | 13746.46 | 0.049 | 0.242 | 0.25±0.31 | 0.15±0.13 |
| 104 | 13783.59 | 0.067 | 0.283 | 0.29±0.53 | 0.14±0.12 |
| 105 | 13900.50 | 0.049 | 0.248 | 0.28±0.25 | 0.19±0.12 |
| 106 | 14457.23 | 0.926 | 0.598 | 0.46±0.30 | 0.45±0.39 |
| 107 | 14517.34 | 0.873 | 0.917 | 0.56±0.35 | 0.57±0.34 |
| 108 | 14913.13 | 0.495 | 0.818 | 0.18±0.15 | 0.16±0.15 |
| 109 | 15066.39 | 0.817 | 0.981 | 7.00±6.58 | 7.51±8.89 |
| 110 | 15278.11 | 0.926 | 0.917 | 0.96±0.94 | 0.98±1.15 |
| 111 | 15486.90 | 0.920 | 0.917 | 0.29±0.28 | 0.28±0.30 |
| 112 | 15970.98 | 0.851 | 0.643 | 0.33±0.42 | 0.37±0.91 |

S8. Correlation of THI x AIDI_H_ of beef bulls of Control and Test groups.


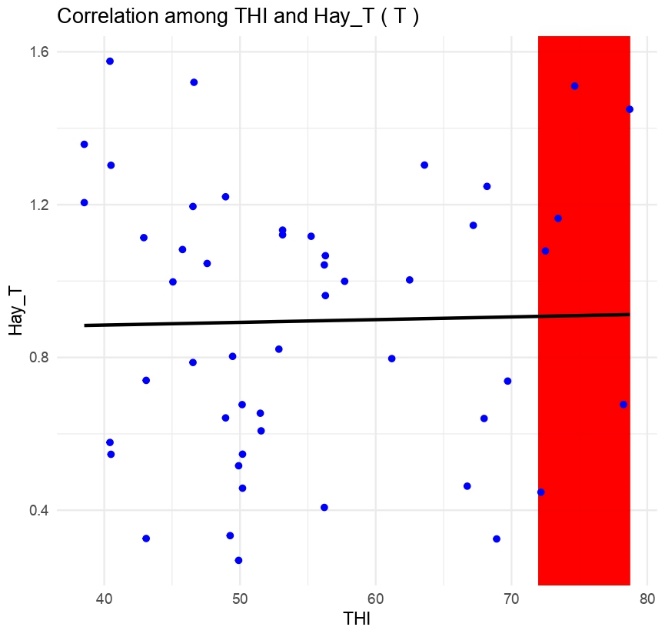

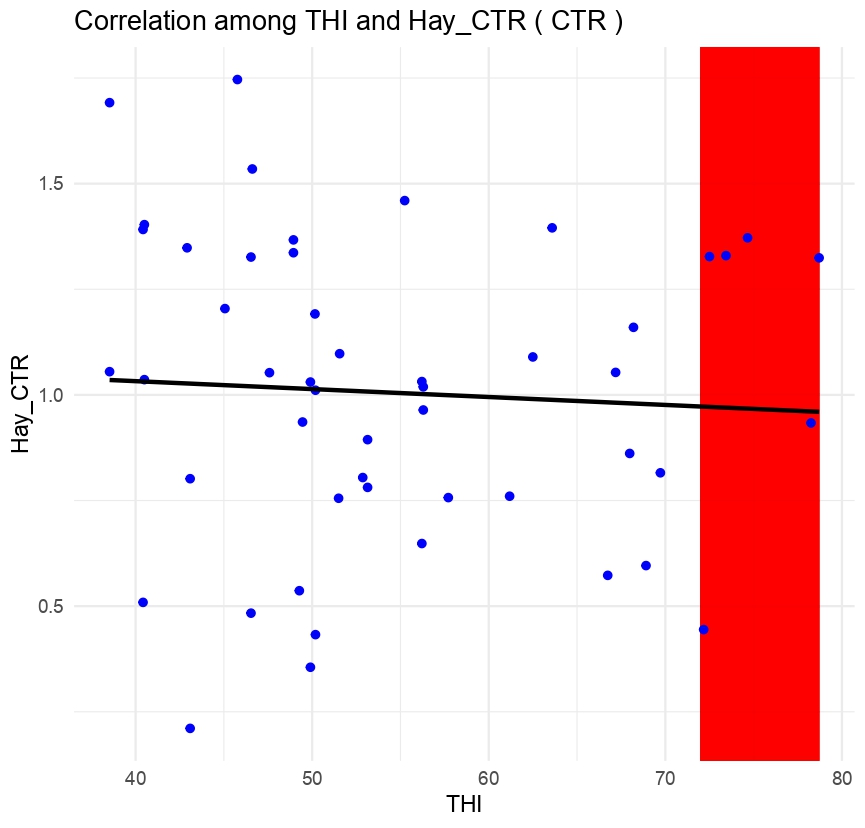


*Red areas indicate THI values above 72, threshold for heat stress in cattle.*

S9. Correlation of THI x AIDI_S_ of beef bulls of Control and Test groups.


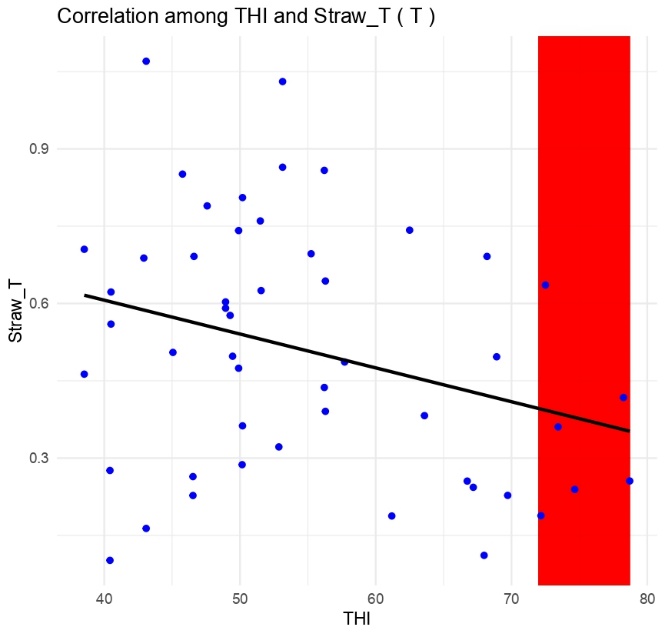

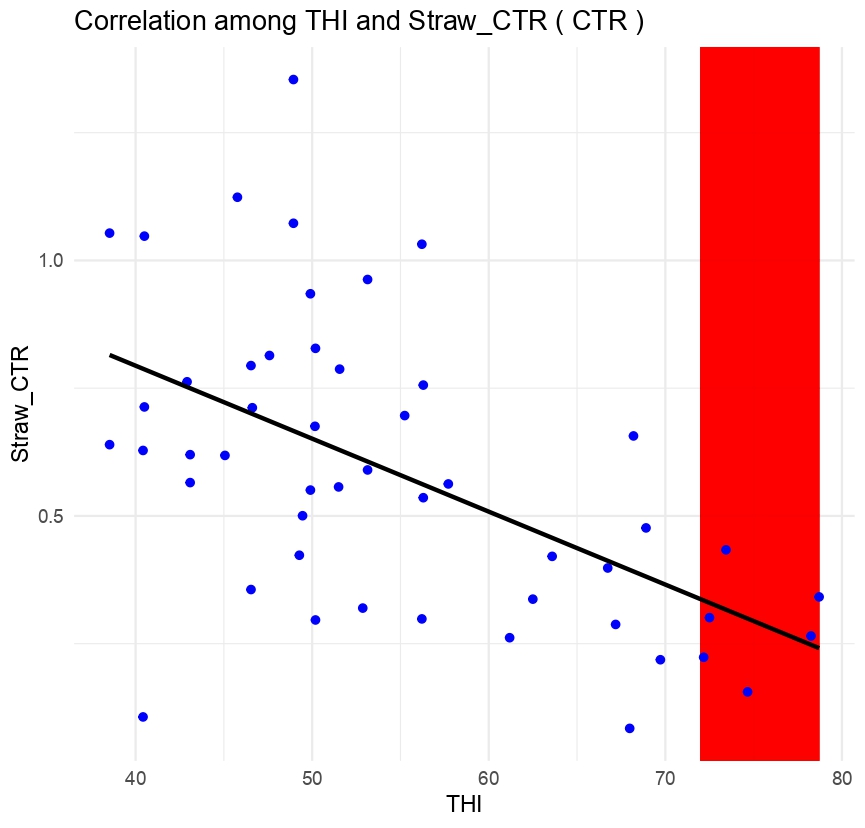


*Red areas indicate THI values above 72, threshold for heat stress in cattle.*

S10. Correlation of THI x AIDI_F_ of beef bulls of Control and Test groups.


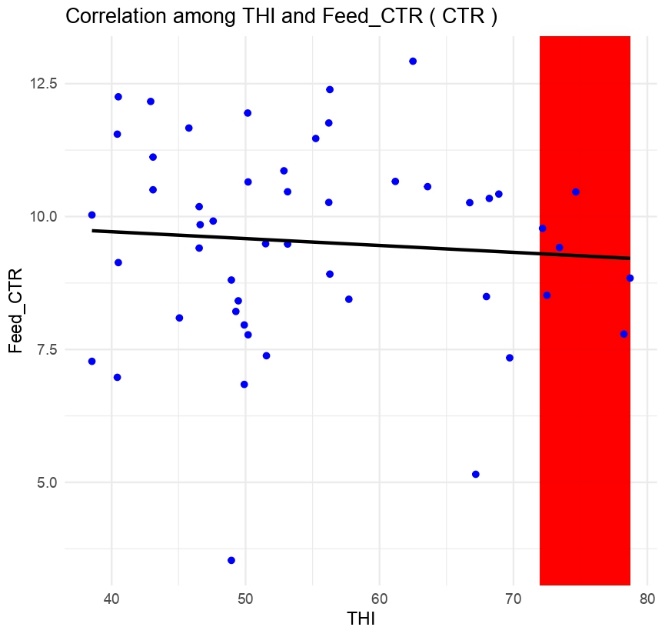

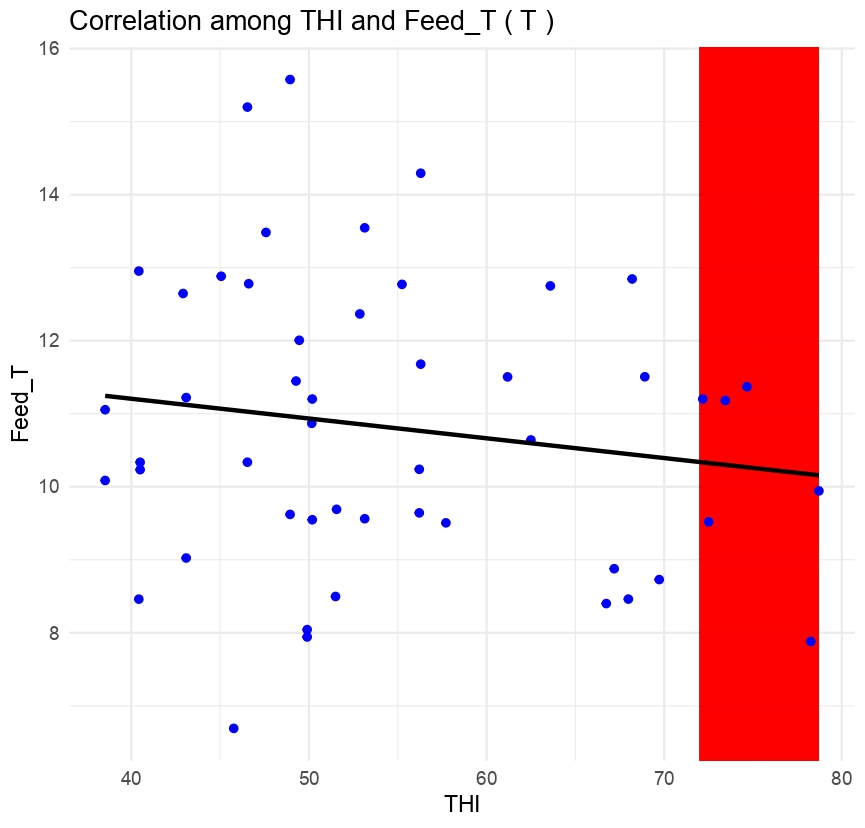


*Red areas indicate THI values above 72, threshold for heat stress in cattle.*
